# Supplementary material for: Dehydration of subducting slow-spread oceanic lithosphere in the Lesser Antilles
Source: Nat Commun. 2017 Jul 10;8:15980. doi: 10.1038/ncomms15980 (PMC5508134; doi:10.1038/ncomms15980)
Supplement: Supplementary Information [file ncomms15980-s1.pdf]

File name: Supplementary Information
Description: Supplementary Figures and Supplementary Tables

File name: Supplementary Movie 1
Description: Rotating animation showing the ray coverage and the subducting slab. Initially we show the P-wave ray paths for shots (gray) and local earthquakes (blue), with the location of the recording stations (black pyramids). The topography of the islands and of the interpreted slab surface are shown for reference. The second part of the animation shows the P-wave velocity anomaly draped on the slab surface. The location of the slab Moho is drawn as a semi-transparent surface assuming a constant 7 km crustal thickness. The outline of the overriding plate Moho is drawn in blue. The contact between the slab surface and the Moho is drawn as a green curve. The location of the backstop is drawn in red. Notice the spatial relationship between the location of seismicity and the local VP minimum at 50 km depth. The volume of the model is the same as shown in Fig. 7. The vertical extent is 162 km. The horizontal extent is 250 km in the arc-parallel direction and 280 km in the arc-perpendicular direction. The animation was prepared using Paraview and encoded using Ffmpeg.

File name: Supplementary Movie 2
Description: Rotating animation for 3D glasses. Same as Supplementary Movie 1, but encoded as a 3D red-cyan stereo anaglyph video to be viewed with common red-cyan 3D glasses.

File name: Peer Review File
Description:

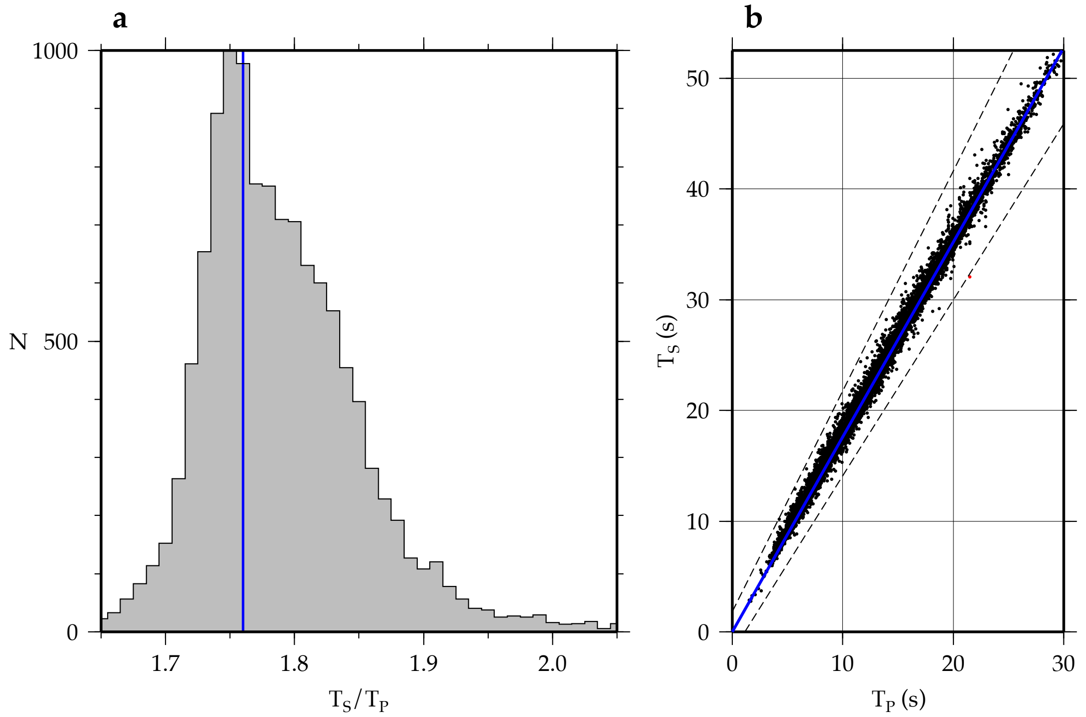

Supplementary Figure 1. Selection of starting V_P/V_S. a) Distribution of ratio of S-wave traveltimes (T_S) to P-wave traveltimes (T_P) for local earthquakes. The blue line marks the mean of the distribution. b) Plot of T_S vs. T_P (Wadati diagram). The blue line represents the linear regression with slope of 1.76. We chose this value to build our starting V_P/V_S model.

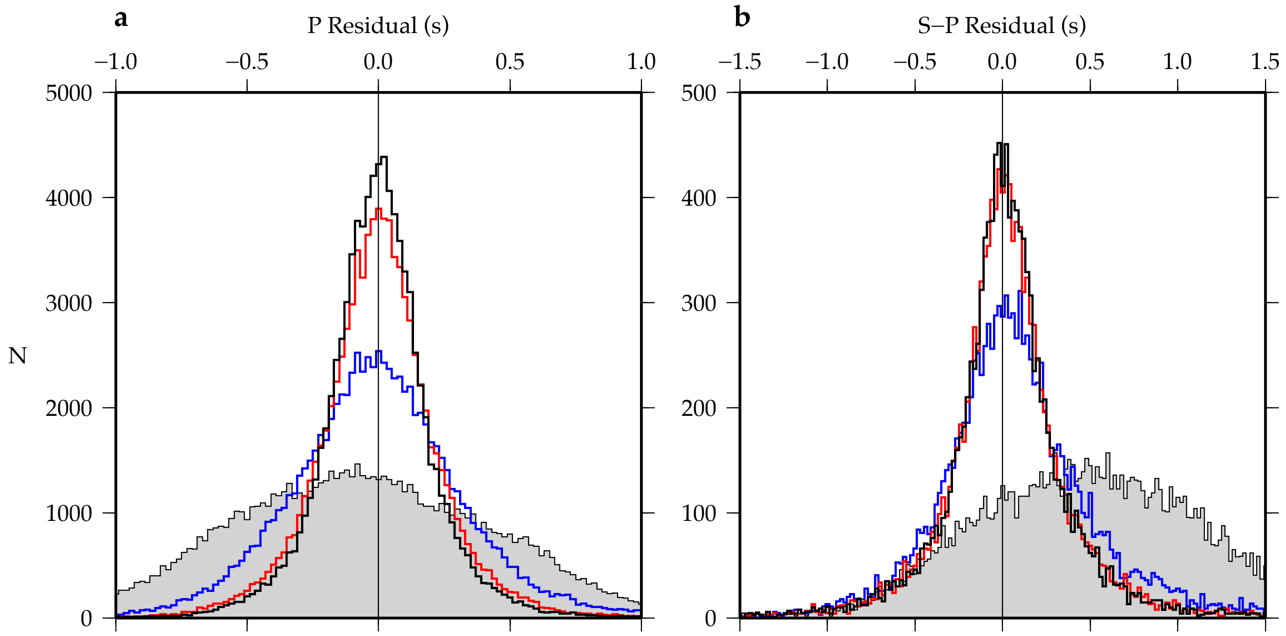

Supplementary Figure 2. Traveltime residuals. a) Histogram of P-wave traveltime residuals for starting model (gray), 40x50 km model (blue), 20x20 km model (red), and 15x15 km model (black). b) Same as a) for S-P traveltime residuals.

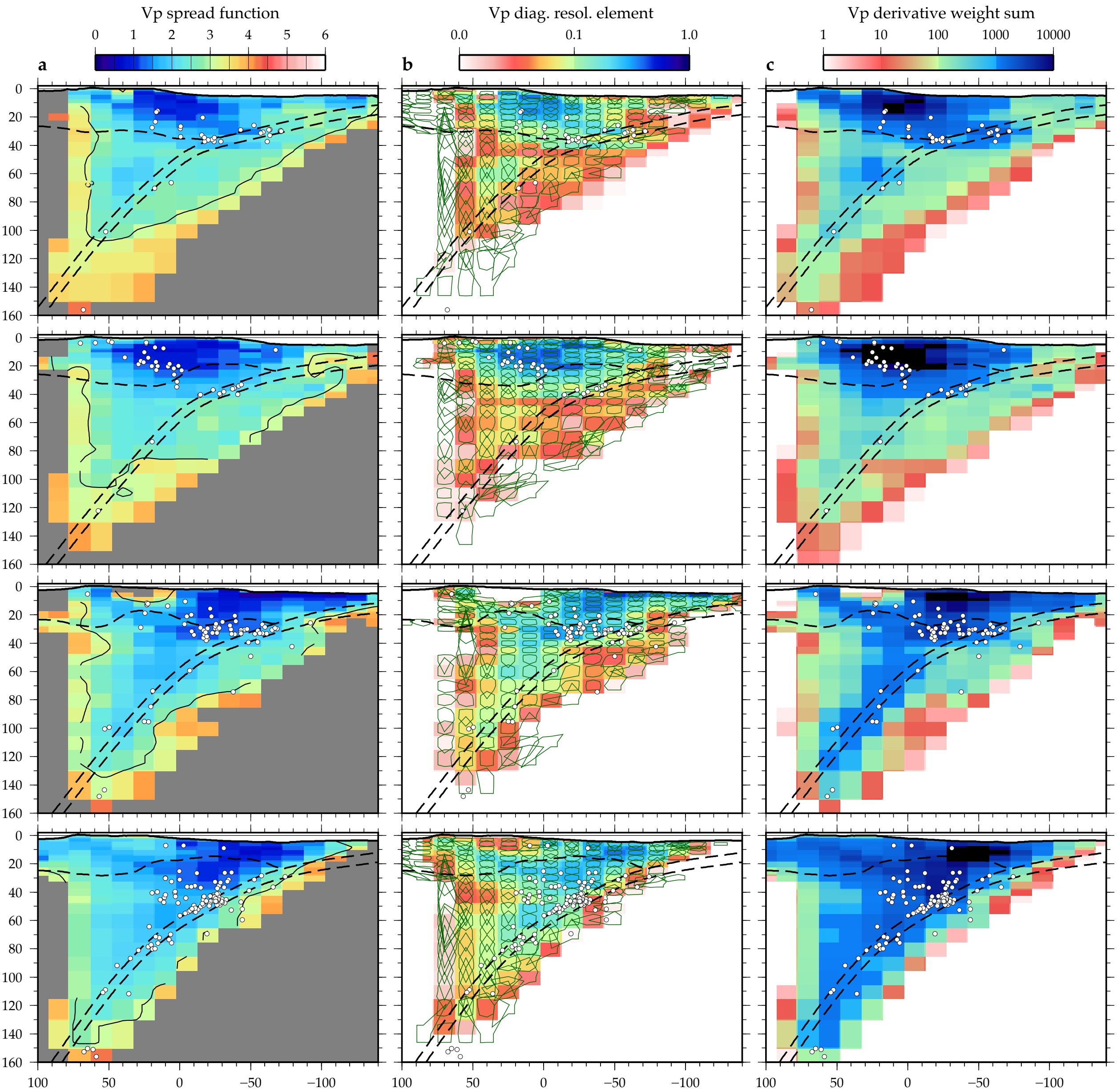

Supplementary Figure 3. *V_P* resolution. a) Spread function along four vertical cross-sections of the model. The profiles are the same as those shown in Fig. 4. b) Diagonal element of resolution matrix (colors) and 70% contours of resolution kernel (contours). c) Derivative weight sum. See Methods for explanation. The dashed lines mark the location of the top and Moho of the slab and the Moho of the overriding plate.

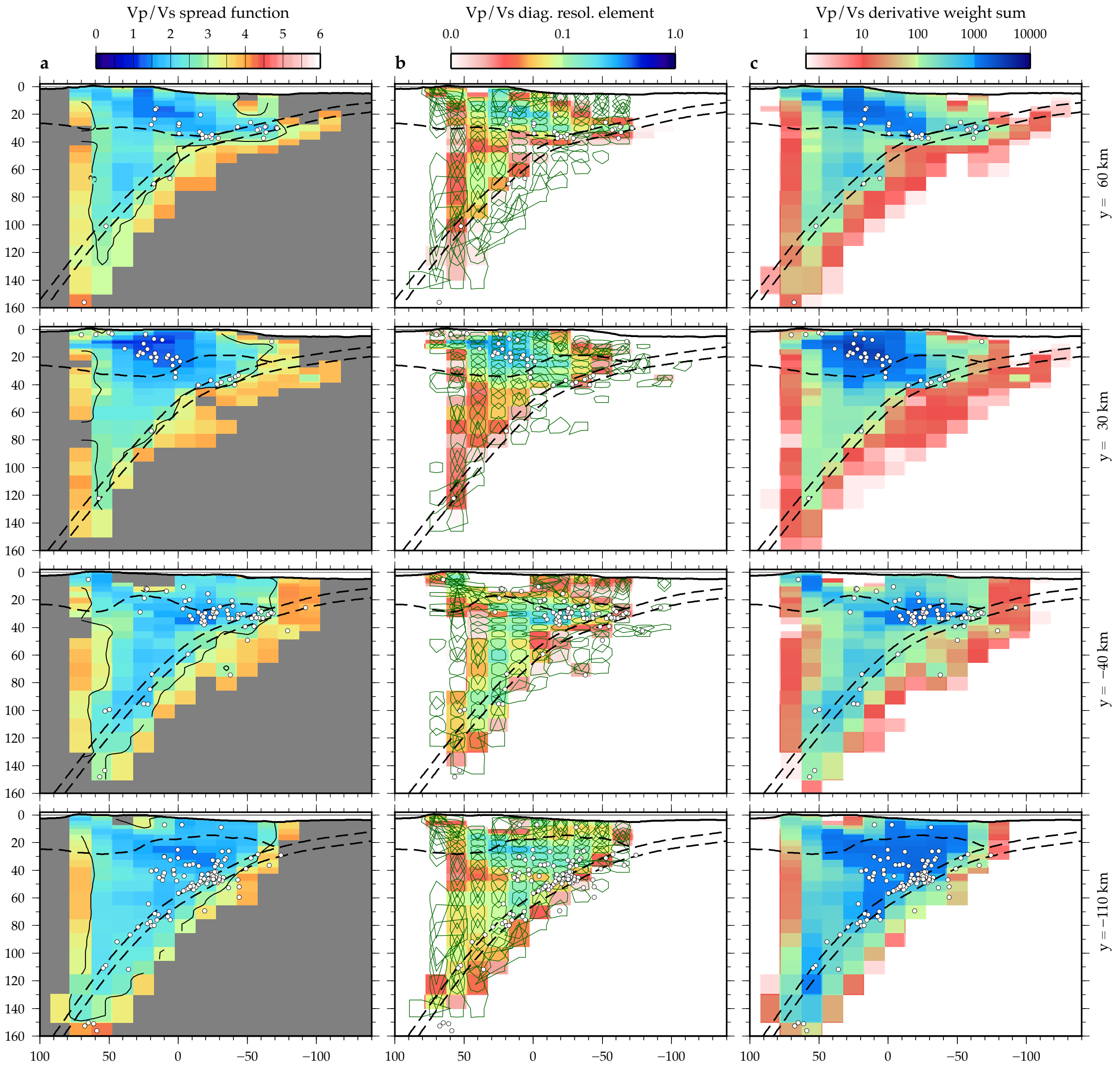

Supplementary Figure 4. V_P/V_S resolution. Same as supplementary Fig. 3, for V_P/V_S model.

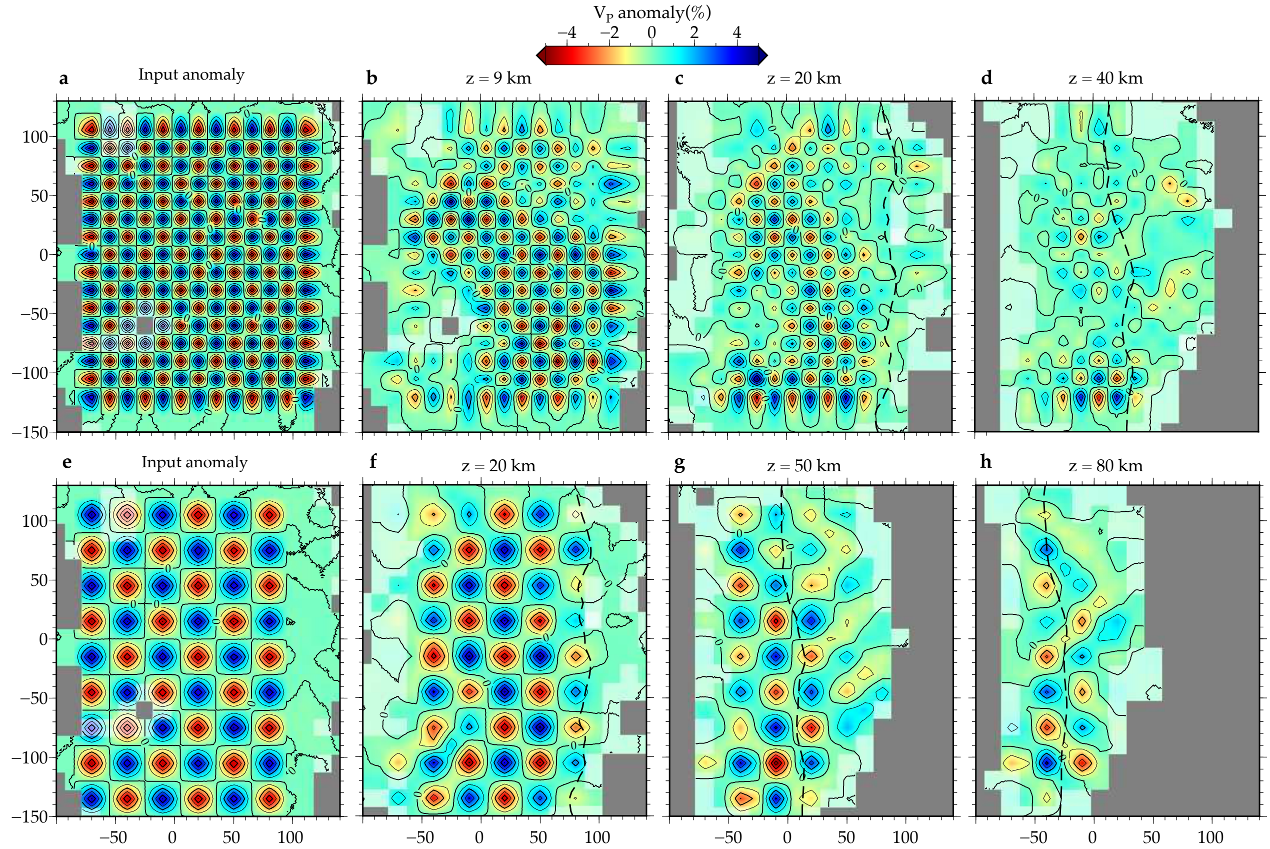

Supplementary Figure 5. V_P checkerboard tests. (a, e) Input and (b, c, d, f, g, h) recovered V_P anomaly for several checkerboard tests. We show the recovery of 15x15 km and 30x30 km anomalies along horizontal sections at depths of 20, 50 and 80 km. The anomalies extend ~30 km in the vertical direction. The dashed black line marks the intersection of the slab surface with the horizontal plane of the section.

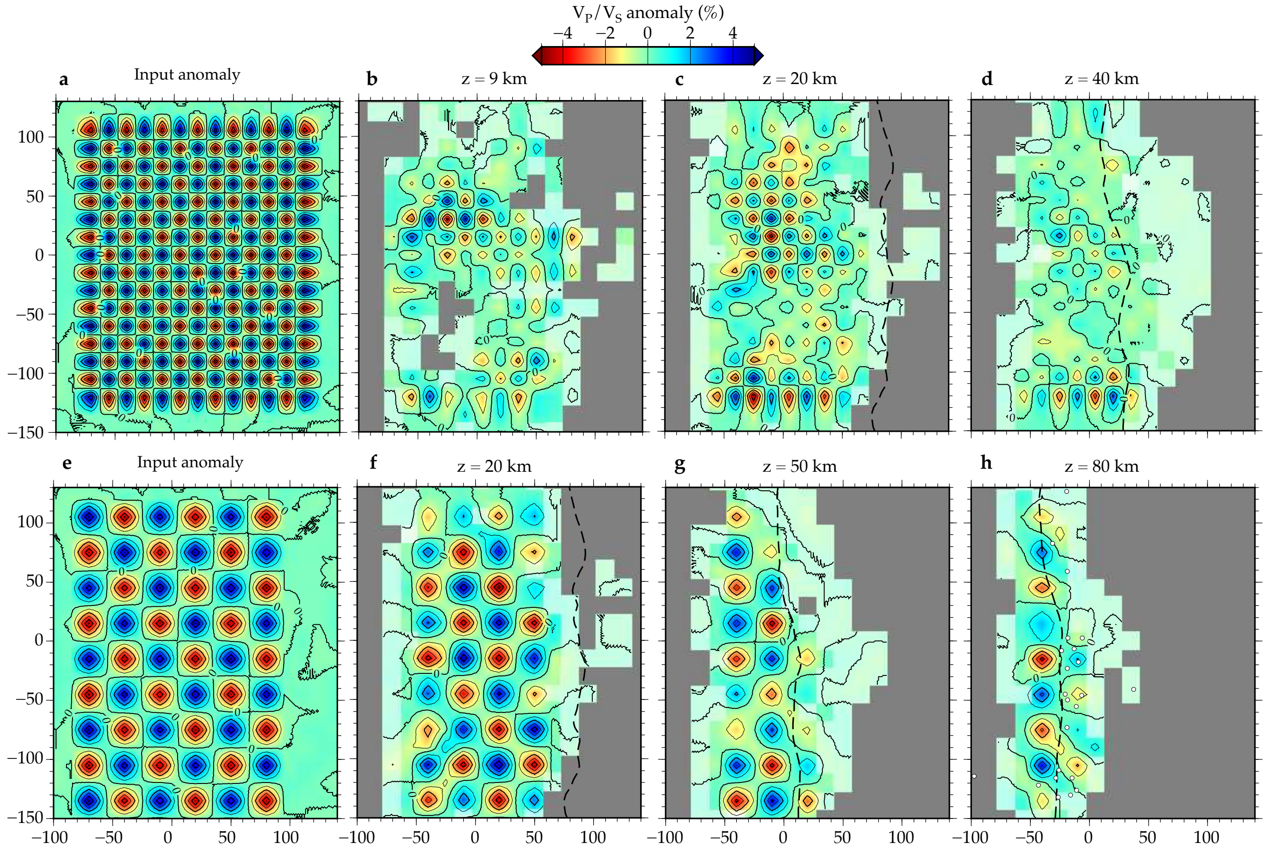

Supplementary Figure 6. V_P/V_S checkerboard tests. Same as Supplementary Fig. 5, for V_P/V_S.

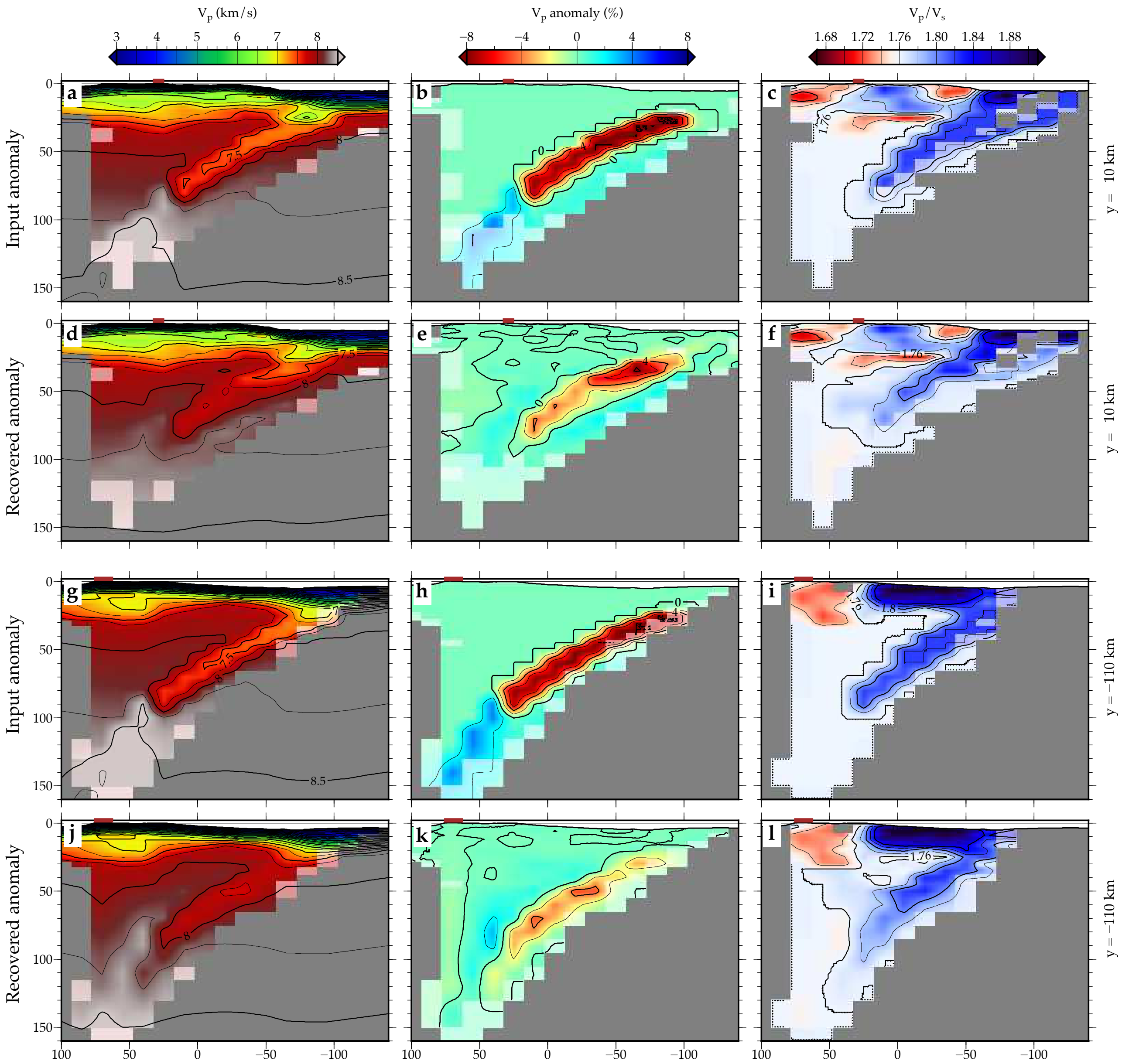

Supplementary Figure 7. Slab anomaly recovery test 1. Vertical sections at different locations in the model showing input and recovered V_P model, V_P anomaly and V_P/V_S. In this test the slab crust low-V_P anomaly terminates at 90 km depth.

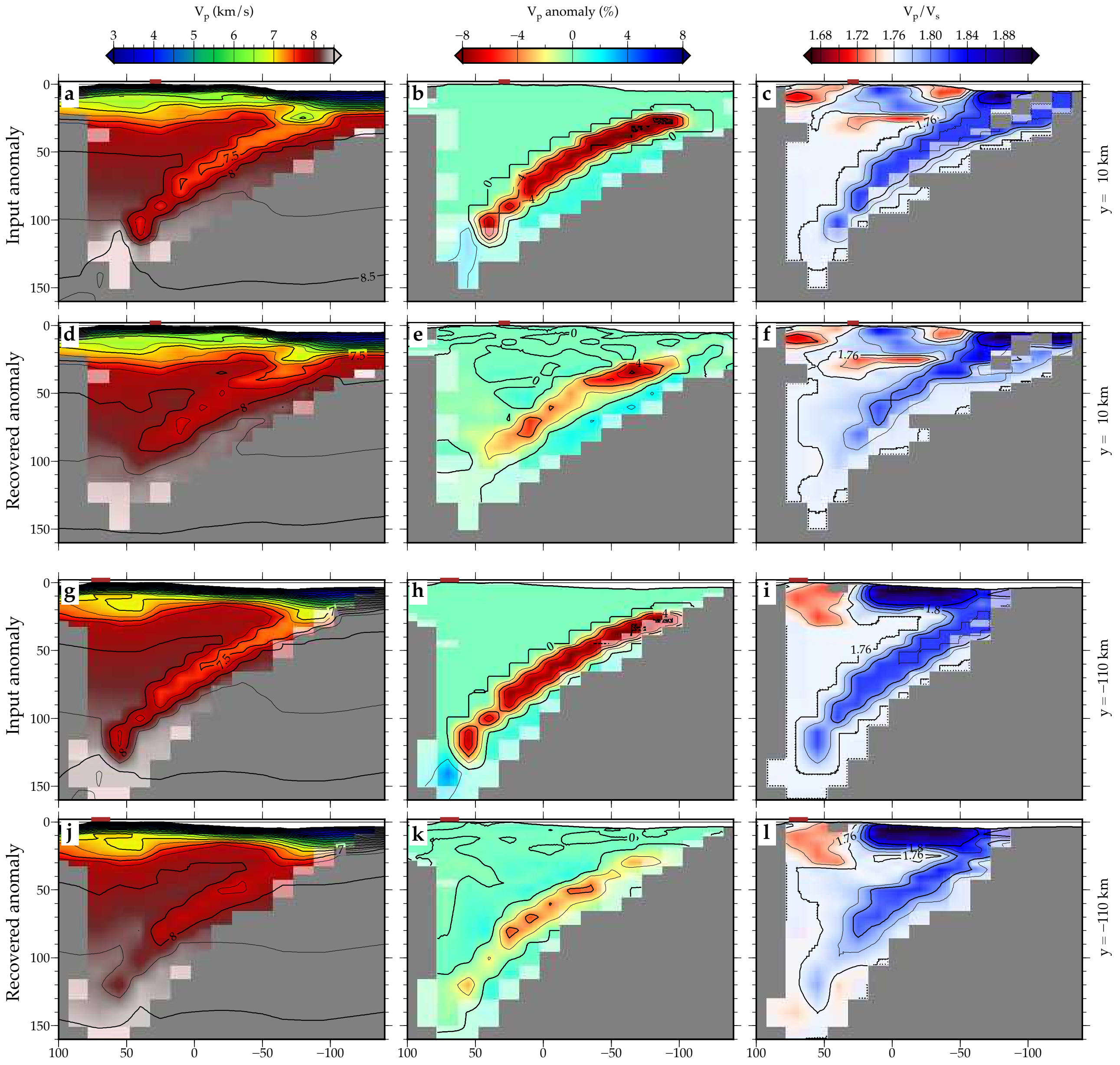

Supplementary Figure 8. Slab anomaly recovery test 2. Vertical sections at different locations in the model showing input and recovered V_P model, V_P anomaly and V_P/V_S. In this test the slab crust low-V_P anomaly terminates at 120 km depth.

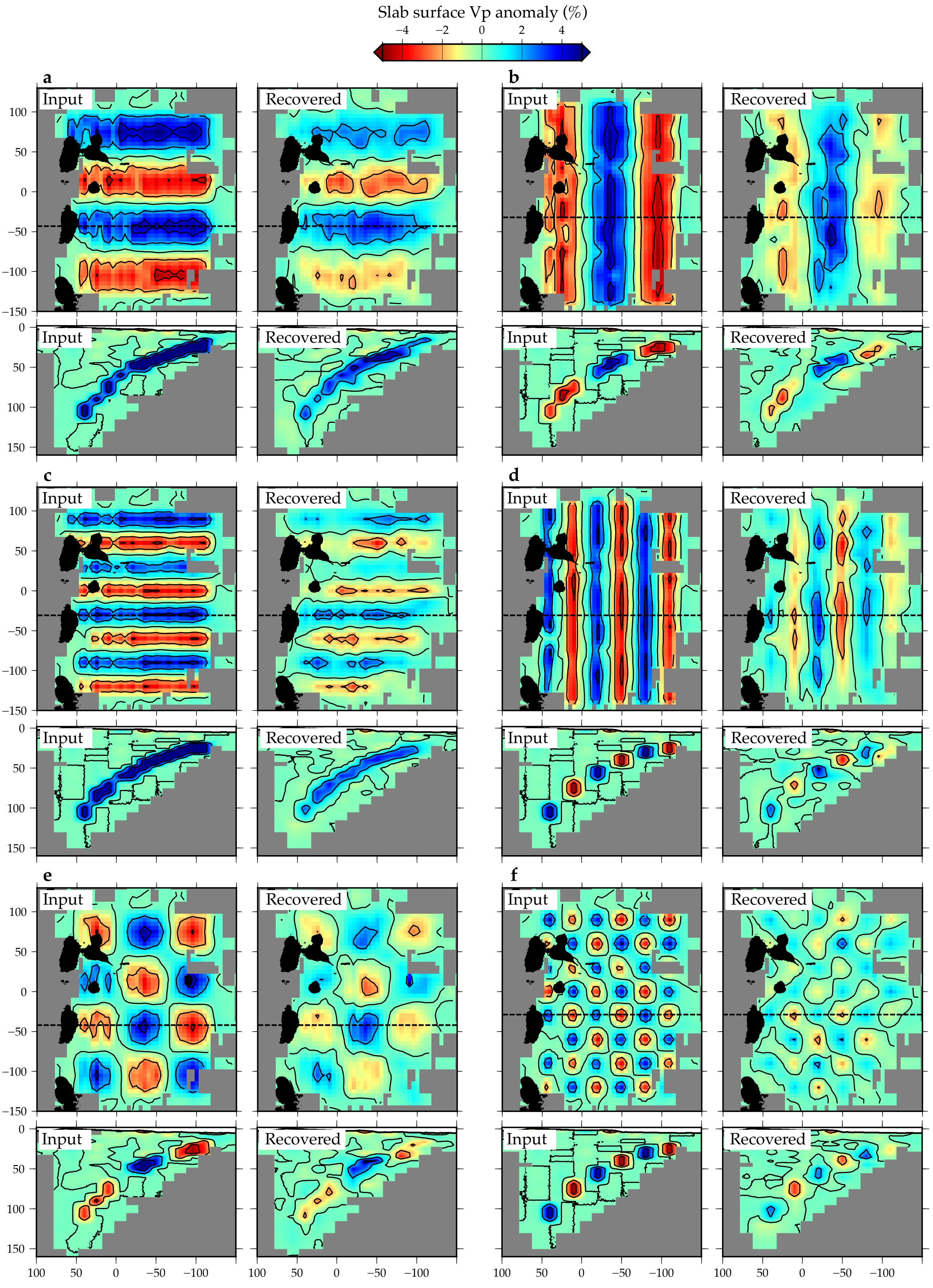

Supplementary Figure 9. Slab V_P checkerboard tests. Input and recovered V_P anomaly for several slab checkerboard tests. We show the anomaly both along the slab top surface and on a vertical section. The islands are colored in black for reference.

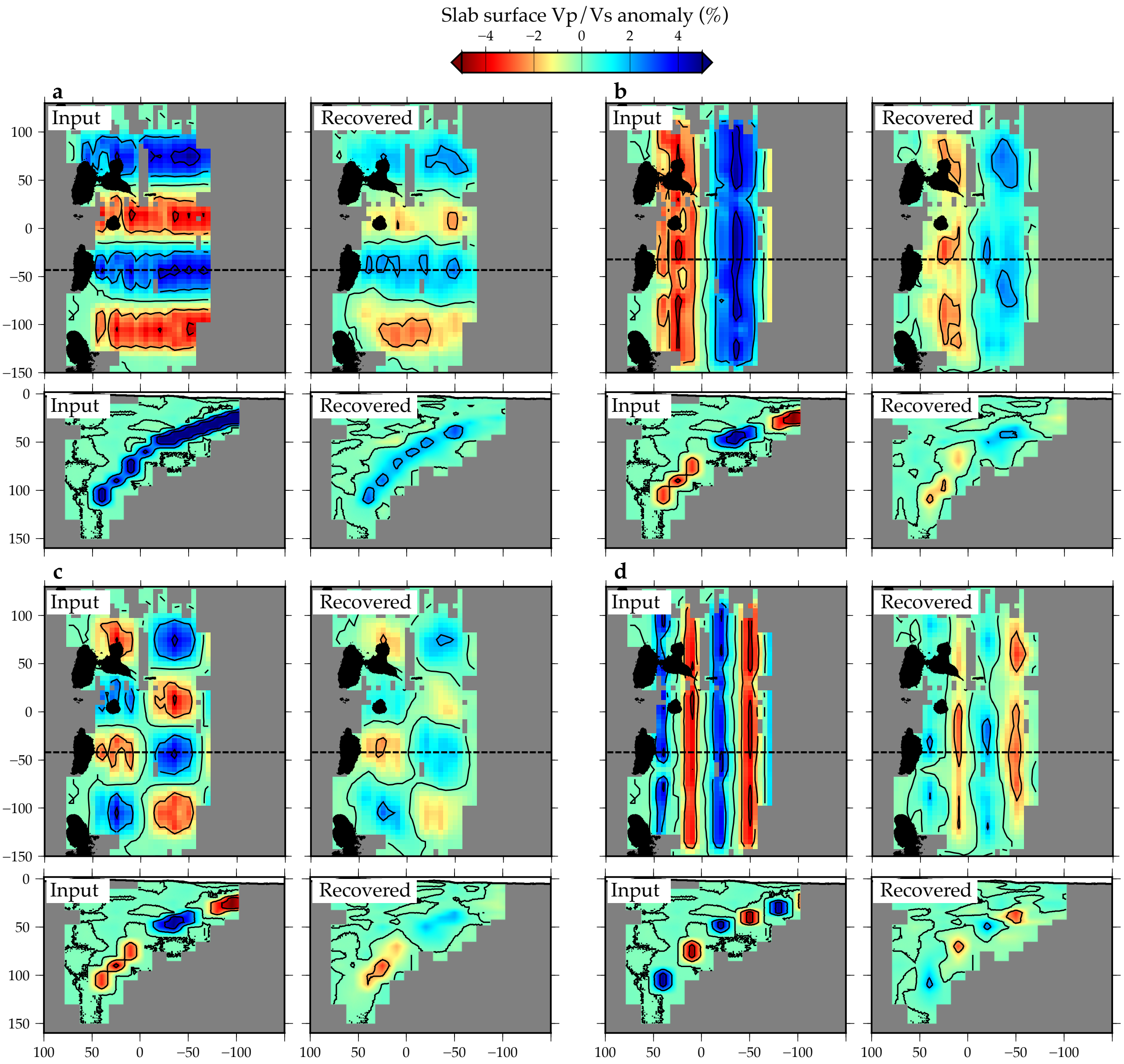

Supplementary Figure 10. Slab V_P/V_S checkerboard tests. Input and recovered V_P/V_S anomaly for several slab checkerboard tests. We show the anomaly both along the slab top surface and on a vertical section. The islands are colored in black for reference.

Inversion step	Minimum horizontal spacing	Minimum vertical spacing	Damping parameters	*RMS* residual (s)			P Data variance		S-P data variance	
				tot	P	S-P	σ^2	%	σ^2	%
1d model	n. a.	n. a.	n. a.	0.73	0.71	0.90				
Starting model	40 km	5 km	n. a.	0.55	0.47	0.88	0.218	100	0.220	100
40x50 km model	40 km	5 km	V_S: 2000<br>V_P/V_S: 2000	0.37	0.35	0.49	0.095	44	0.130	59
20x20 km model	20 km	5 km	V_P: 500<br>V_P/V_s: 500	0.28	0.26	0.40	0.048	22	0.071	32
15x15 km final model	15 km	3 km	V_P: 250<br>V_P/V_S: 125	0.26	0.24	0.39	0.038	17	0.067	30

Supplementary table 1: Summary of inversion parameters and residual statistics.
